# From Pruritus to Bullae: Recognizing Furosemide-Induced Bullous Pemphigoid in an Elderly Male Patient

**DOI:** 10.7759/cureus.76856

**Published:** 2025-01-03

**Authors:** Nicole Johnsen, Sarah Goldgar

**Affiliations:** 1 Department of Dermatology, David Geffen School of Medicine at the University of California Los Angeles, Los Angeles, USA; 2 Department of Medicine, Division of General Internal Medicine, University of California Los Angeles, Los Angeles, USA

**Keywords:** drug induced bullous pemphigoid, dupilumab, elderly population, furosemide, refractory pruritus, tense bullae

## Abstract

Bullous pemphigoid (BP) is an autoimmune blistering disorder characterized by tense bullae and severe generalized pruritus. However, atypical presentations in elderly patients may involve localized pruritus prior to the development of bullae, which can obscure the diagnosis and delay appropriate treatment. We present a case of BP induced by furosemide in an 83-year-old male patient with congestive heart failure. The patient initially experienced persistent pruritus on the bilateral arms and back, which emerged approximately three months after initiating furosemide therapy. This pruritus was initially attributed to xerosis. Subsequently, erythematous papules developed in these areas, raising suspicion for atypical scabies, although they did not respond to permethrin treatment. A shave biopsy revealed subepidermal bullous disease with numerous eosinophils, and direct immunofluorescence confirmed the diagnosis of BP, showing linear IgG and granular C3 deposition at the basement membrane zone. The papules then evolved into tense bullae, and serologic testing supported the diagnosis with elevated IgG titers and positive BP180 antibodies. Furosemide was discontinued as the likely trigger. Initial treatment with prednisone led to improvement; however, bullae reappeared upon tapering the medication. Further management with doxycycline and topical triamcinolone resulted in enhanced improvement. Ultimately, biweekly dupilumab injections effectively controlled the symptoms. This case underscores the diagnostic challenges of BP, particularly in the elderly, where isolated pruritus may precede the appearance of visible bullous lesions. Clinicians should maintain a high index of suspicion for BP in elderly patients with unexplained pruritus, especially when standard treatments are ineffective. Additionally, this case highlights the importance of considering drug-induced BP, with furosemide recognized as a potential trigger.

## Introduction

Bullous pemphigoid (BP) is a rare autoimmune blistering disease with an annual incidence of approximately 12-13 cases per million, as reported in studies conducted in Switzerland and Germany [1,2]. It primarily affects individuals aged 65-75, with a slight male predominance [3].The pathogenesis involves IgG autoantibodies targeting BP180 and BP230, hemidesmosomal proteins crucial for dermo-epidermal junction (DEJ) stability [4,5]. Binding of these autoantibodies disrupts the integrity of the DEJ, triggering an inflammatory response. This leads to edema and inflammatory cell accumulation at the DEJ, ultimately resulting in bullae formation [6]. Clinically, BP typically presents with large, tense, serous or hemorrhagic bullae overlying erythematous, urticarial, eczematous or seemingly normal skin. These lesions are often distributed on the trunk, extremities, or intertriginous areas [3], and generally evolve into crusted erosions that heal without scarring [7]. However, BP can exhibit considerable phenotypic variability, posing significant diagnostic challenges.

In approximately 20% of cases, particularly among older adults [1], BP may begin with a non-bullous prodromal phase, characterized by pruritus (100%), erythematous urticarial lesions (52.3%), excoriations (22.7%), and papules or nodules (20.5%) [8]. In around 9.8% of these cases, pruritus can persist for weeks to months before progressing to bullae [8,9]; however, for many patients, these pruritic lesions may persist without ever developing into bullae [10]. Diagnosing BP during this prodromal phase remains challenging, as its clinical presentation may resemble other conditions, such as xerosis, drug reactions, prurigo, eczema, dermatitis, or scabies [3,11]. In elderly patients with persistent, unexplained pruritus over eczematous or urticarial lesions, BP should be considered in the differential diagnosis after excluding common etiologies [10,12]. Early diagnosis is crucial, as prompt treatment enables effective disease control and improved prognosis [13]. If undiagnosed, BP often follows a chronic course with intermittent remissions and exacerbations [14].

Diagnosing BP typically involves a combination of histopathology and direct immunofluorescence (DIF) analysis of the affected skin. Histopathological examination using hematoxylin and eosin staining reveals detachment at the DEJ along with a mixed inflammatory infiltrate rich in eosinophils, as well as varying numbers of lymphocytes and neutrophils [15,16]. DIF testing demonstrates linear deposition of IgG and complement component 3 at the DEJ [17]. Additionally, serological tests, such as indirect immunofluorescence on monkey esophagus and human salt-split skin, can help distinguish BP from other blistering disorders. BP is identified by linear DEJ on the monkey esophagus substrate, with a sensitivity of 73%. Furthermore, BP exhibits "roof" binding on human salt-split skin, which is highly specific and differentiates it from conditions like pemphigus vulgaris, which shows "floor" binding on the same substrate [18].

After confirming the diagnosis, management of BP typically involves topical steroids, systemic corticosteroids, and/or doxycycline. Localized disease often responds well to potent topical corticosteroids, such as clobetasol. However, more extensive involvement may necessitate systemic corticosteroids, like prednisone [13]. For refractory cases that do not respond adequately to these initial treatments, clinicians may need to consider utilizing immunomodulatory agents (e.g., intravenous immunoglobulin, dapsone), immunosuppressants (e.g., azathioprine, mycophenolate mofetil, methotrexate, cyclosporine, and cyclophosphamide), or biologics (dupilumab, rituximab, omalizumab) [19].

While understanding effective management strategies is crucial, recognizing the underlying etiology of BP is equally important. Although the primary cause of BP is not fully understood, the initial presentation and exacerbation of the disease have been associated with various triggers, such as drugs, burns, vaccines, infections, trauma, surgical procedures, ultraviolet light radiation, and radiation therapy [15,20]. Notably, drug-induced BP has been linked to a range of medications, including antibiotics, beta-blockers, non-steroidal anti-inflammatory drugs, diuretics, and gliptins. Among these, gliptins (e.g., sitagliptin, alogliptin) pose the highest risk, although diuretics, particularly loop diuretics (e.g., furosemide, bumetanide), thiazides, and potassium-sparing diuretics (e.g., spironolactone), are also common triggers, especially in elderly patients [15]. This report discusses a case of furosemide-induced BP in an elderly male patient who initially presented with prodromal pruritus, followed by the development of erythematous papules and eventually the presence of bullae.

## Case presentation

**Figure 1:**
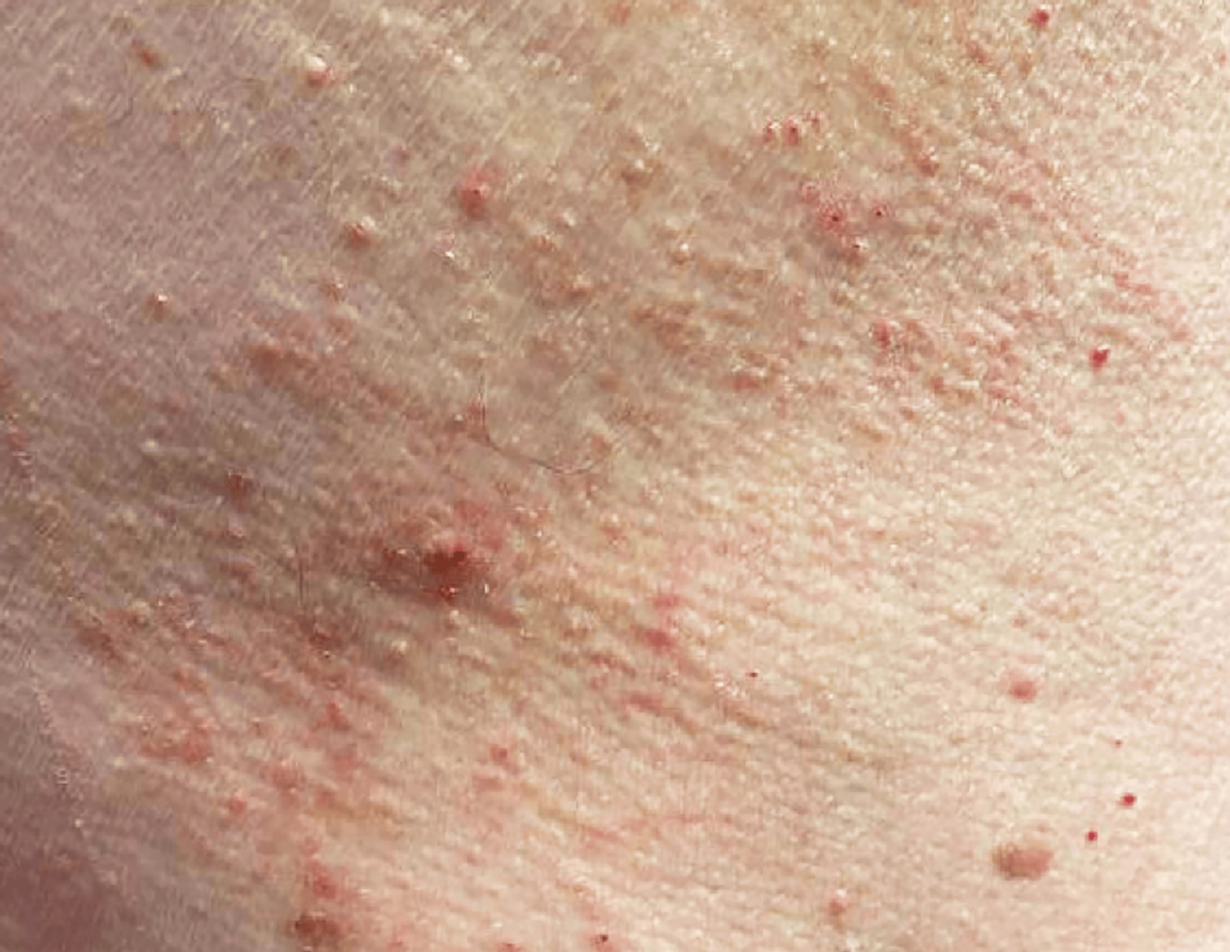
Initial presentation of bullous pemphigoid Erythematous papules are shown on the back, accompanied by the onset of pruritus.

An 83-year-old male patient with a history of congestive heart failure presented to his primary care physician with persistent bilateral pruritus affecting his arms and back. This symptom emerged approximately three months after starting furosemide therapy (20 mg three times a day). Initially, given the lack of rash or other associated symptoms, the pruritus was attributed to xerosis, with differential diagnoses including atopic dermatitis and allergic contact dermatitis. Despite the prescription of triamcinolone cream (0.025% PRN (pro re nata; as needed)) and loratadine (10 mg in the evening), the patient's symptoms persisted. As the pruritus intensified, erythematous papules developed on his bilateral arms and back, as shown in Figure 1, highlighting the lesions on his back. Given this unusual presentation and intense pruritus, scabies was considered; however, treatment with 5% permethrin was ineffective, leading to a referral for dermatological evaluation.

**Figure 2:**
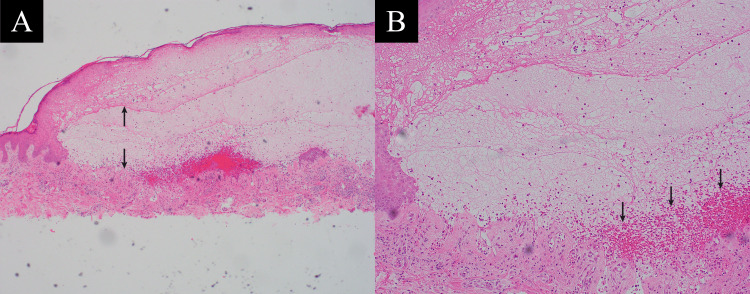
Histopathologic features of bullous pemphigoid on hematoxylin and eosin stain (A) Low-power view (40x magnification) of a hematoxylin and eosin-stained section from a shave biopsy of an erythematous papule on the back. A subepidermal blister is evident, defined by separation at the dermoepidermal junction, with a cavity containing a mixed inflammatory infiltrate. The black arrows indicate the borders of the epidermis and dermis, highlighting the large cavity between them. (B) High-power view (100x magnification) provides greater detail of the subepidermal blister, revealing inflammatory infiltrates composed predominantly of eosinophils and lymphocytes within the blister cavity and upper dermis. The surrounding dermis exhibits edema and prominent eosinophilic infiltration (indicated by black arrows), findings characteristic of bullous pemphigoid.

At the dermatology clinic, a shave biopsy of an erythematous papule on the back was performed, revealing histopathological findings characterized by a significant eosinophilic infiltrate consistent with subepidermal bullous disease (Figure 2). DIF studies demonstrated linear deposition of IgG and granular complement component C3 along the basement membrane zone. 

**Figure 3:**
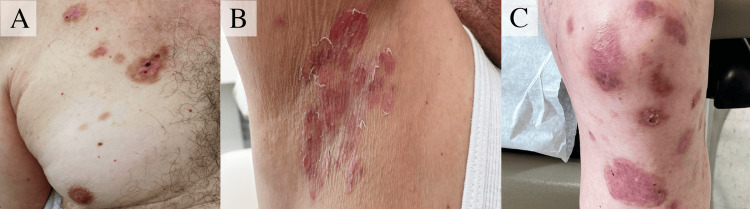
Clinical progression of bullous pemphigoid (A) Healing erythematous erosions on the chest, (B) axilla, and (C) knee, demonstrating areas of post-inflammatory hyperpigmentation following the resolution of tense bullae.

The erythematous papules subsequently evolved into tense bullae and erosions affecting the patient's arms, legs, back, chest, axilla, and groin. Healing erosions on the patient's chest, axilla, and knee are depicted in Figure 3. The integration of the clinical presentation, histological findings, and DIF results led to a confirmed diagnosis of BP. Serological testing further corroborated this diagnosis, revealing elevated IgG titers (monkey esophagus: 1:640; human split skin: 1:1280) and positive antibodies against BP180.

Recognizing the strong association between furosemide and the onset of BP, furosemide was discontinued as a likely trigger. The patient was then started on systemic prednisone (20 mg daily) as first-line therapy, accompanied by clobetasol ointment for the open lesions (0.05% twice daily PRN). Initial clinical improvement was observed; however, the bullae reappeared after tapering the prednisone from 20 mg to 10 mg over two weeks. To address these recurrent symptoms, oral doxycycline (100 mg twice daily) and topical triamcinolone (0.1% twice daily PRN) were introduced, along with continued low-dose oral prednisone (10 mg) and clobetasol ointment (0.05% twice daily PRN), resulting in further clinical benefits. Ultimately, dupilumab injections (300 mg, every other week) were incorporated into the treatment regimen, achieving effective control of symptoms, providing significant relief from pruritus, and resolving the bullae.

## Discussion

This case highlights the diagnostic challenges of BP in elderly patients, who often present with atypical features differing from the general population. While BP typically manifests as tense bullae on erythematous or urticarial skin, elderly individuals frequently exhibit a prodromal phase characterized by pruritic lesions or may present only pruritus in the absence of rash. This early presentation can easily be mistaken for other common dermatological conditions, complicating timely diagnosis and treatment. In our case, the patient's presentation began with isolated pruritus, progressing to erythematous papules and ultimately resulting in bullae.

In addition to these diagnostic challenges, recognizing the underlying triggers of BP is crucial for effective management, as discontinuing the causative agent can lead to significant improvements in patient outcomes. Therefore, we recommend that primary care physicians maintain a heightened awareness of diuretic-induced BP, particularly in elderly patients, as these medications are commonly prescribed for cardiovascular conditions and represent a common trigger for BP in this population.

Lastly, this case underscores the importance of a multidisciplinary approach in managing complex BP cases. Referrals to dermatology are vital for accurate diagnosis and individualized treatment strategies. Effective collaboration between primary care providers and dermatologists not only facilitates immediate management but also enhances long-term care, ultimately improving the quality of life for patients with BP.

## Conclusions

This case highlights the diagnostic challenges of BP in elderly patients, where isolated pruritus may precede the development of bullous lesions. Clinicians should maintain a high index of suspicion for BP, especially when standard treatments for pruritus are ineffective. Incorporating histopathological analysis, DIF, and serological testing, often through dermatology consultation, into the diagnostic approach is essential for addressing unexplained pruritus in this patient population. Moreover, recognizing diuretic-induced BP in the elderly is crucial, as many common diuretics, including loop diuretics, thiazides, and potassium-sparing diuretics, are frequent triggers in this patient population.
